# Phytoplankton stoichiometry along the salinity gradient under limited nutrient and light supply

**DOI:** 10.1093/plankt/fbae031

**Published:** 2024-06-11

**Authors:** Iris D S Orizar, Sonja I Repetti, Aleksandra M Lewandowska

**Affiliations:** Tvärminne Zoological Station, Faculty of Biological and Environmental Sciences, University of Helsinki, J.A. Palmenin 260, 10900 Hanko, Finland; Tvärminne Zoological Station, Faculty of Biological and Environmental Sciences, University of Helsinki, J.A. Palmenin 260, 10900 Hanko, Finland; Tvärminne Zoological Station, Faculty of Biological and Environmental Sciences, University of Helsinki, J.A. Palmenin 260, 10900 Hanko, Finland

**Keywords:** Baltic Sea, salinity, carbon accumulation, phytoplankton stoichiometry, environmental filtering

## Abstract

Ongoing climate warming alters precipitation and water column stability, leading to salinity and nutrient supply changes in the euphotic zone of many coastal ecosystems and semi-enclosed seas. Changing salinity and nutrient conditions affect phytoplankton physiology by altering elemental ratios of carbon (C), nitrogen (N) and phosphorus (P). This study aimed to understand how salinity stress and resource acquisition affect phytoplankton stoichiometry. We incubated a phytoplankton polyculture composed of 10 species under different light, inorganic nutrient ratio and salinity levels. At the end of the incubation period, we measured particulate elemental composition (C, N and P), chlorophyll *a* and species abundances. The phytoplankton polyculture, dominated by *Phaeodactylum tricornutum*, accumulated more particulate organic carbon (POC) with increasing salinity. The low POC and low particulate C:N and C:P ratios toward 0 psu suggest that the hypoosmotic conditions highly affected primary production. The relative abundance of different species varied with salinity, and some species grew faster under low nutrient supply. Still, the dominant diatom regulated the overall POC of the polyculture, following the classic concept of the foundation species.

## INTRODUCTION

Climate warming is associated with changes in the chemistry and circulation of the oceans, altering light and nutrient regimes (Arteaga *et al.*, 2014), leading to changes in phytoplankton community structure and productivity (Henson *et al.*, 2021). Simultaneously, rising global temperatures and changing precipitation patterns affect the global water cycle (Durack *et al.*, 2012), resulting in salinity fluctuations that affect phytoplankton physiology and the allocation of essential elements (nitrogen and phosphorus) in their cells. Our understanding of salinity effects on phytoplankton traits is still limited compared to other environmental factors, such as temperature and pCO_2_. We have little information on how resources are allocated when the phytoplankton communities are exposed to hypo- and hyperosmotic conditions and how these changes can affect essential cellular activities such as photosynthesis (Kirst, 1989; Mohammed and Shafea, 1992; Hernando *et al.*, 2015).

Numerous studies have investigated the impact of limited resources on phytoplankton composition and elemental stoichiometry (Huisman and Weissing, 2001; Sterner and Elser, 2002; Litchman *et al.*, 2004; Dickman *et al.*, 2006; Klausmeier *et al.*, 2008; Yoshiyama *et al.*, 2009; Tanioka and Matsumoto, 2017). A phytoplankton community under nutrient-replete conditions tends to have a carbon:nitrogen:phosphorus (C:N:P) ratio of 106:16:1 (Redfield, 1958), but research indicates that individual species differ in their elemental composition (Geider and La Roche, 2002), which can be attributed to changes in the available nutrient supply, irradiance, temperature, and pCO_2_ (Klausmeier *et al.*, 2004; Galbraith and Martiny, 2015; Tanioka and Matsumoto, 2017; Yvon-Durocher *et al.*, 2017; Velthuis *et al.*, 2022). Therefore, C:N:P proportions in natural phytoplankton assemblages often deviate from the Redfield ratio, varying spatially and temporarily (Martiny *et al.*, 2013; Arteaga *et al.*, 2014; Bonachela *et al.*, 2016). Generally, phytoplankton communities in the subtropical oceans have higher C:N and C:P ratios (C:nutrients) due to low nutrient availability (Galbraith and Martiny, 2015; Tanioka and Matsumoto, 2020), while the communities in the colder regions have a low N:P ratio from increased P-rich molecules, such as ribosomal RNA, in the cold-adapted phytoplankton (Toseland *et al.*, 2013; Daines *et al.*, 2014).

Light availability is another critical factor controlling phytoplankton composition and growth. For example, freshwater and estuarine phytoplankton communities grew slower under low-light conditions (Lionard *et al.*, 2005), but different phytoplankton have species-specific light requirements, and some taxa are better adapted to low-light conditions (Brauer *et al.*, 2012; Edwards *et al.*, 2015). Light availability directly affects the functioning of photosynthetic apparatus, but it also affects phytoplankton dynamics and elemental composition (Litchman and Klausmeier, 2001; Striebel *et al.*, 2008; Brauer *et al.*, 2012). In general, increased irradiance results in higher photosynthetic carbon fixation (Sterner *et al.*, 1997; Dickman *et al.*, 2006; Striebel *et al.*, 2008; Tanioka and Matsumoto, 2020), but under strong salinity stress, the positive effect becomes negligible due to the failure of the photosynthetic machinery (Kirst, 1989; Coupel *et al.*, 2015; Hernando *et al.*, 2015).

Our understanding of how light and nutrients regulate phytoplankton growth, coexistence, trophic transfer efficiency and ecosystem productivity is progressing, but how these effects interact with salinity remains unresolved. Research in the last decade has primarily focused on the effects of warming on phytoplankton production and stoichiometry (Biermann *et al.*, 2015; Yvon-Durocher *et al.*, 2017). The consequences of climate-driven salinity change garnered less attention, and we still need to establish how salinity affects phytoplankton resource acquisition and storage (Cunillera-Montcusí *et al.*, 2022). On the individual level, salinity changes can affect metabolic rates of algal cells due to osmotic adjustments done through the production of more osmolytes (hyperosmotic conditions) or active removal of solutes from the cell (hypoosmotic conditions), influencing algal stoichiometry (Kirst, 1989). On the community level, research suggests that salinity has little effect on the phytoplankton resource use efficiency, but it might cause phytoplankton dominance shifts (Olli *et al.*, 2023) resulting in changes in the phytoplankton community response to resource limitation (Marcarelli *et al.*, 2006).

Salinity is a strong environmental filter, structuring phytoplankton communities and potentially leading to local extinctions when conditions become unfavorable, especially for species with narrow salinity tolerance (Flöder *et al.*, 2010; Cloern *et al.*, 2017). Therefore, phytoplankton diversity is reduced in brackish ecosystems, such as estuaries and coastal waters, with a minimum species richness falling between 7 and 8 psu (Olli *et al.*, 2019). The majority of phytoplankton species die off when exposed to stressful salinity conditions (below or above species tolerance threshold) due to a lack of pathways to synthesize osmolytes in freshwater lineages or an inability to downregulate osmolyte production in marine lineages (Olli *et al.*, 2023).

For our study, we composed an artificial phytoplankton assemblage of 10 species isolated from the Baltic Sea and exposed this polyculture to different salinity, nutrient supply and light conditions. Selected species had different set of traits (cell size, shape, type of exoskeleton and motility) and are co-occurring in the Baltic Sea. Predicted freshening of the Baltic Sea (Meier *et al.*, 2012; Vuorinen *et al.*, 2015; Lehmann *et al.*, 2022) is expected to put additional pressure on species with higher salinity optima, potentially leading to local extinctions, reorganization of community structure and disruption of ecosystem functions, including carbon storage capacity.

With our experiment, we aimed to answer the following questions:

1. How does salinity influence the elemental composition of a phytoplankton assemblage under different levels of light and nutrient conditions?
2. To what extent do changes in species relative abundances explain the variation in particulate C:N:P ratios of the phytoplankton community along the salinity gradient?

We expected that salinity and resource supply would affect phytoplankton community structure, stoichiometry and consequently biomass production.

## MATERIALS AND METHOD

### Preparation of phytoplankton cultures

Ten monoalgal cultures (co-occurring Baltic Sea species) from the FINMARI Culture Collection at the Tvärminne Zoological Station, Finland (Table I), were used to create an artificial phytoplankton assemblage. Species were selected to maximize the diversity of traits, which can be affected by salinity change (e.g. cell size, shape, type of exoskeleton). The monocultures were maintained under controlled conditions before the experiment (salinity = 5 psu, temperature = 16°C, nutrient supply: F/2 media, light setting: 130 μmol photons m^−2^ s^−1^, 16:8 h light:dark cycle). For the polyculture experiment, species monocultures were mixed in equal proportions based on their *in vivo* chlorophyll a fluorescence. Because cellular chlorophyll a content varies between species affecting their fluorescence, it cannot be assumed that the species in the polyculture had equal initial cell density, but the community composition at the start of the experiment was the same among the experimental units and the treatments. This procedure might have affected carbon and nutrient partitioning between the species in the polyculture, but not the observed treatment effects.

**Table I:** *List of phytoplankton species used in the experiment*

Species	Strain	Phylum	Cell cover	Cell size (μm)^*^	Geometric shape^*^	Remarks
*Phaeodactylum tricornutum*	TV 335	Bacillariophyta	Silica frustules	15–27	Half parallelepiped	Form multiple morphotypes
*Diatoma tenuis*	DTTV B5	Bacillariophyta	Silica frustules	40–80	Parallelepiped	Form zig zag colonies
*Skeletonema marinoi*	C1407	Bacillariophyta	Silica frustules	13	Cylinder	Chain forming
*Alexandrium ostenfeldii*	AOF 0940	Miozoa	Cellulose thecate	24–40	Rotational ellipsoid	PSP-toxin producer
*Levanderina fissa*	GFF 1101	Miozoa	Athecate	5–74	Flattened ellipsoid	
*Kryptoperidinium foliaceum*	KFF 0901	Miozoa	Cellulose thecate	30–50	Sphere-25%	Large eyespot
*Rhodomonas marina*	CRYPTO 07B1	Cryptista	Periplast	20	Cone + half sphere	
*Diacronema lutheri*	TV 03	Haptophyta	Naked	7–9	Flattened ellipsoid	
*Monoraphidium* sp.	TV 70	Chlorophyta	Naked	6–60	Spindle	
*Synechococcus* sp.	TV 65	Cyanobacteria	Naked	1–3	Sphere	Not documented/observed to fix nitrogen

Note: ^*^cell size and geometric shape are based from Olenina *et al.*, 2006

### Experimental set-up

A three-factorial experiment was designed by combining different light (photosynthetic photon flux density = 10 and 130 μmol photons m^−2^ s^−1^), inorganic nutrient ratio (N:P = 16, 80, 2 and 10), and salinity (0, 5, 12 and 24 psu) conditions with three replicates for each treatment, resulting in 96 experimental units (volume: 540 mL unit^−1^) randomly distributed in a temperature-controlled room (16°C). We used six-channel sunlight spectrum LED lamps (Aquarius plant, Aqua Medic GmbH) programmed to have a 16:8 hours light–dark cycle. The different salinity levels were achieved by adding Aquarium System Sea Salt (Instant Ocean Spectrum) (15, 20, 30 and 35 psu) or MilliQ water (5 psu) to prefiltered (0.2 μm), sterilized seawater (6 psu) collected offshore of the Tvärminne Zoological Station at Storfjärden (59° 51.318’ N, 23° 15.796′ E). MilliQ water was used to mimic the freshwater condition. Desired initial nutrient conditions were achieved by modifying the nitrate and phosphate components of F/2 media, while the trace metals and vitamin components were retained (Guillard and Ryther, 1962). The N:P ratio of 16 (NO_3_ = 80 μM, PO_4_ = 5 μM) represents a balanced nutrient supply defined by Redfield (Redfield, 1934, 1958) and is referred to as nutrient-replete conditions throughout the manuscript. The N:P ratio of 10 represents nutrient-depleted conditions, where both nutrients are depleted (NO_3_ = 10 μM, and PO_4_ = 1 μM). The N:P ratios of 80 (NO_3_ = 80 μM, PO_4_ = 1 μM) and 2 (NO_3_ = 10 μM, PO_4_ = 5 μM) simulate P and N-depleted conditions, respectively. All treatment ratios refer to the initial conditions. Phosphorus and nitrogen uptake at the end of the incubation period were confirmed by measurements of the residual dissolved inorganic nutrient concentrations (Fig. S3). Both, N:P = 16 and 10 treatments were nitrogen and phosphorus depleted by the end of the experiment. All nutrient ratios throughout the manuscript are provided as molar ratios.

### Sampling

**Fig. 1:**
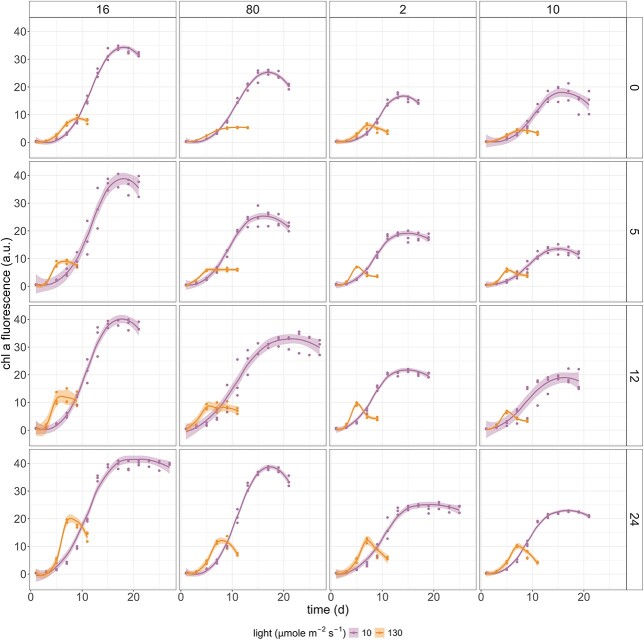
Growth curves of the experimental phytoplankton community under different salinities and inorganic nutrient ratios. From left to right: N:P = 16, 80, 2 and 10. From top to bottom: salinity = 0, 5, 12 and 24 psu. Curves are based on the *in vivo* chl-*a* fluorescence measurements, and the smoothing functions were generated using ggplot2 with shaded area representing 95% confidence intervals (smoothing method = *loess*).

The initial phytoplankton biomass of every experimental unit was measured 12 hours after the culture preparation using chl-*a* fluorescence as a proxy (t_0_). After that, fluorescence was monitored every other day at the same time of the day to follow the phytoplankton growth and estimate when the cultures reached the stationary phase. Each experimental treatment was terminated when all replicates reached the stationary phase of growth, therefore the incubation period ranged from 9 to 27 days depending on the treatment (Fig. 1).

At the end of the incubation period, samples were collected for measurement of dissolved nutrients (NH_4_, NO_x_, PO_4_ and Si), particulate organic carbon (POC), nitrogen (PON) and phosphorus (POP), chl-*a* concentration and cell density. Samples for microscopy were preserved with acidified Lugol’s iodine solution. The abundance of *Synechococcus* sp. was estimated from samples preserved with formaldehyde (2% final concentration) and stored at −80°C until analysis.

### Chlorophyll a and Pheophytin a

Samples were collected on a glass fiber filter (GF/F Whatman). The chl-*a* was extracted using 94% ethanol for 24 hours. The extracted chl-*a* fluorescence was measured using a Cary Eclipse Fluorescence Spectrophotometer (excitation = 430 nm, and emission = 670 nm). Subsamples were acidified using HCl (0.008 M final concentration) to determine pheophytin *a* (phe-*a*) concentration (Parker *et al.*, 2016). The chl-*a* values were corrected by subtracting the corresponding phe-*a* concentrations.

### Dissolved and particulate nutrients

To measure dissolved inorganic nutrients, samples were filtered through cellulose acetate filters (0.2 μm pore size) and analyzed using a continuous flow autoanalyzer (AAII) following the Hansen and Koroleff protocol (Hansen and Koroleff, 2007).

The POC, PON and POP samples were collected on acid-washed, pre-combusted GF/F filters. The POC/N samples were dried at 60°C for 24 h and analyzed using an elemental analyzer (Vario MicroCube, Elementar, Germany). The POP samples were combusted at 450°C for 4 h and analyzed colorimetrically following the protocol of Koistinen *et al.* (Solorzano and Sharp, 1980; Koistinen *et al.*, 2017).

### Species abundance and growth rate

Cell counting was performed following the Utermöhl method (Utermöhl, 1958) using a DM IRB inverted microscope (Leica, Wetzlar, Germany) with the aid of LAS X software. Counting was guided by HELCOM proceedings (Lund *et al.*, 1958; HELCOM, 2018).

The *Synechococcus* sp. samples were analyzed using a BD Accuri C6 plus flow cytometer. The cell density was calculated using C6 plus analysis software (BD Biosciences).

The growth rate was calculated using the *in vivo* chl-*a* fluorescence measured during the incubation. The following equation was used:


(1) \begin{equation*} \mu =\kern0.75em \frac{\ln{f}^1-\ln{f}^0}{\Delta t} \end{equation*}

Where: μ = growth rate

f^1^ = *in vivo* chl-*a* fluorescence at the end of the incubation period

f^0^ = *in vivo* chl-*a* fluorescence at t_0_

Δt = incubation period (days)

The contribution of each species to the total final biomass of the polyculture was calculated using the biovolumes obtained from the monocultures (Figs S1 and S4). Equations from Olenina et al. (Olenina *et al.*, 2006) were used to calculate the respective biovolume and carbon content of each species.

### Statistical analyses

All statistical analyses were performed using R statistical software ver. 4.2.0 (R Core Team, 2022). The packages *Factoextra* (Kassambara and Mundt, 2020) and *ggplot2* (Wickham, 2016) were used to visualize the results.

Multiple factor analysis (MFA) (Pages, 2002) was used to test which group of variables (treatments, phytoplankton community, elemental stoichiometry, residual nutrients) contributed the most to the overall variability among the experimental units. The package *FactoMineR* (Le *et al.*, 2008) was used to analyze the results.

Generalized linear models (GLMs) were used to determine the effects of light (two levels) and nutrient conditions (four levels) along the salinity gradient on the phytoplankton elemental stoichiometry, growth rate (μ), extracted chlorophyll-*a* and maximum chlorophyll-a fluorescence (Fluo_max_). Salinity was treated as a continuous explanatory variable, while light and inorganic nutrient ratios were treated as categorical factors. Models were fitted using maximum likelihood estimation, and model selection was performed using the Akaike information criterion. The interaction terms are reported whenever they were significant. Variance partitioning was calculated from McFadden’s R^2^.

## RESULTS

### Phytoplankton growth and community composition

The polycultures reached the stationary phase of growth between 9 and 27 days, depending on the treatment (Fig. 1). Light intensity had a significant effect on the growth rate (μ) and maximum chlorophyll *a* fluorescence (Fluo_max_) (Fig. S2). The growth rate under low-light intensity (μ = 0.18–0.30 day^−1^) was, on average, 1.8 times slower than under high-light conditions (μ = 0.32–0.64 day^−1^) (GLM: ß = 1.80, SE = 1.34, *P* < 0.001), regardless of salinity and inorganic nutrient ratio of the medium. On the other hand, the polycultures under low-light intensity reached 1.2 times higher maximum chl-*a* fluorescence (Fluo_max_ = 13.39–41.18 a.u.) than under high light intensity (Fluo_max_ = 3.43–19.75 a.u.) (GLM: ß = 0.23, SE = 0.47, *P* < 0.001). Nutrient depletion led to a decline of Fluo_max_ by nearly half compared to nutrient-replete conditions (GLM: ß = 0.46, SE = 0.714, *P* < 0.001). Salinity solely had no significant effect on μ and Fluo_max_ (*P* > 0.5), and its effect on phytoplankton growth was dependent on light and nutrient conditions (Fig. S2).

The residual inorganic nutrient concentrations (NO_x_ = NO_3_ + NO_2_, NH_4_, and PO_4_) were generally under the detection limit by the end of the incubation period at higher salinities, but not at lower salinity conditions (Fig. S3a). Nitrogen was not completely taken up under P-depleted conditions (N:P = 80), PO_4_ was reduced by at least 90% across all treatments. Low salinity conditions were characterized by higher ammonia (NH_4_) concentrations compared to high salinity treatments, regardless of light and nutrient supply. Silicate utilization increased with salinity only under N:P = 16 (Fig. S3a).

The total chl-*a* concentration increased with salinity, was higher under low-light intensity, and decreased in response to nutrient limitation (Fig. S3b). The chl-*a* concentration was positively correlated with Fluo_max_, POC and PON, and negatively correlated with μ (Fig. S5).

**Fig. 2:**
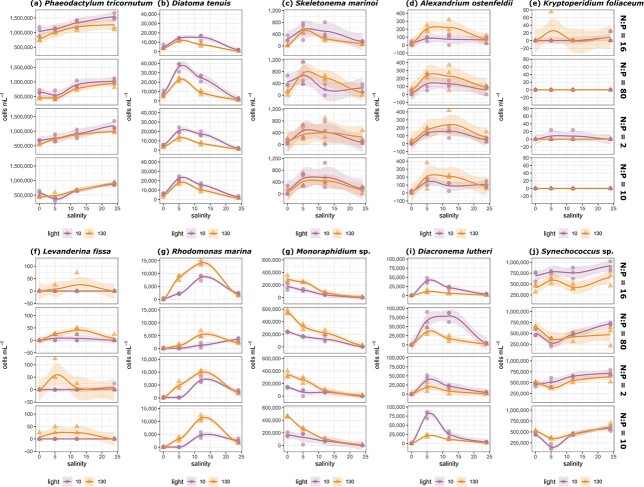
Final cell density of the 10 phytoplankton strains (a–j) along the salinity gradient under different combinations of light (circle = 10, triangle = 130 μmol photons m^−2^ s^−1^), and nutrient (N:P molar ratios, top-bottom) conditions. Smoothing function was generated using the *ggplot2* package (method = *loess*) and shaded area represents 95% confidence intervals.

The diatom *Phaeodactylum tricornutum* dominated the polycultures by the end of the incubation period in all of the treatments (Fig. S4), in terms of cell density (Fig. 2) and biovolume (Fig. S4), with increasing values under higher salinity conditions (Fig. 2). The final abundance of *Synechococcus* sp. also increased with salinity and was positively correlated with *P. tricornutum* (Fig. S6). In contrast, the final abundance of *Monoraphidium* sp. increased under freshwater conditions and was negatively correlated with *P. tricornutum*. Other species reached the highest final cell density at intermediate salinity levels (Fig. 2).

### Sources of variation between experimental units

**Fig. 3:**
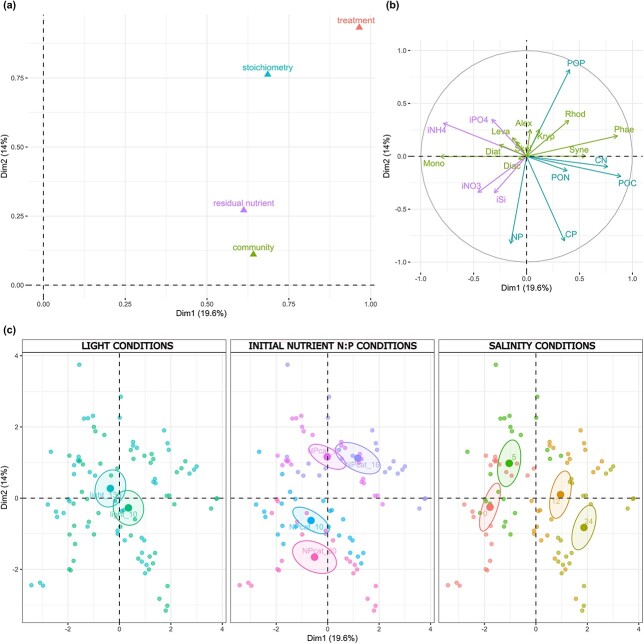
Results of the MFA. (a) Contribution of variables sorted by group. (b) Contribution of the quantitative variables to the variation in the first two dimensions. (c) Clustering of observations by light intensity (left panel), inorganic nutrient ratio (center) and salinity conditions (right). Phae = *Phaeodactylum tricornutum*, Diat = *Diatoma tenuis*, Skel = *Skeletonema marinoi*, Alex = *Alexandrium ostenfeldii*, Kryp = *Kryptoperidinium foliaceum*, Leva = *Levanderina fissa*, Rhod = *Rhodomonas marina*, Mono = *Monoraphidium* sp., Diac = *Diacronema lutheri*, Syne = *Synechococcus* sp., residual nutrients: iPO4 = phosphate, iNO3 = nitrate, iNH4 = ammonium, iSi = silica, particulate nutrient: POC = particulate organic carbon, PON = particulate organic nitrogen, POP = particulate organic phosphate, molar ratios: CN = carbon:nitrogen, CP = carbon:phosphorus, NP = nitrogen:phosphorus.

MFA identified experimental treatment (salinity × light × nutrient ratio) as the main source of variation, followed by POC:PON:POP stoichiometry. Residual inorganic nutrient concentrations and community composition contributed less to the variability between experimental units (Fig. 3a). The first two dimensions explained 33% of the variation, sorting the observations by salinity along the first dimension, and by nutrient ratio treatment along the second dimension (Fig. 3c).

POC was positively correlated with dimension 1, contributing 10% to the variation along this axis, while POP was positively correlated with dimension 2 contributing 12%. N:P ratio was negatively correlated with dimension 2, contributing 12% to the variation (Fig. 3b).

The MFA also confirmed species salinity preferences. The diatom *P. tricornutum,* which had the highest relative abundance under 24 psu, was located on the opposite side to the green algae *Monoraphidium* sp. thriving under 0 psu conditions (Fig. 3). Most species were distributed at the center of the plot (Fig. 3b) indicating their preference for salinity levels within the range 5–12 psu.

The abundance of *P. tricornutum* was highly correlated with POC and POP (Fig. S7). The inorganic nutrient ratio and salinity conditions explained 81% of the variation in *P. tricornutum* cell density. In turn, *P. tricornutum* cell density explained 48% of the variation in POC, and 28% of the variation in POP (Fig. S8).

### Particulate organic carbon, nitrogen and phosphorus

**Fig. 4:**
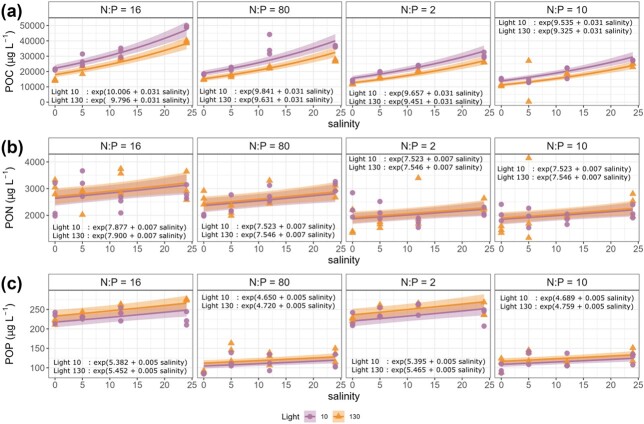
(a) POC, (b) PON and (c) POP concentration at different combinations of salinity (0, 5, 12 and 24 psu), light (circle = 10, triangle = 130 μmol photons m^−2^ s^−1^) and nutrient ratio (N:P molar ratio = 16, 80, 2 and 10, left to right panel) conditions. The lines are predicted values from the GLMs with 95% confidence intervals.

GLMs were formulated to analyze the effect of salinity, light and nutrient conditions on particulate organic concentration and carbon, nitrogen and phosphorus ratios of the polycultures. POC, PON and POP concentrations increased with salinity (Fig. 4). Salinity explained 41% of the variation in POC, 6% of the variation in PON and 1% of the variation in POP. Nutrient treatments explained the largest proportions of variation in PON and POP (34 and 87%, respectively, Fig. S8). Light intensity significantly affected POC (6% variation) and POP (1%) but not PON. The POC was 0.81 times lower and POP 1.04 times higher under high light conditions compared to low-light conditions.

**Fig. 5:**
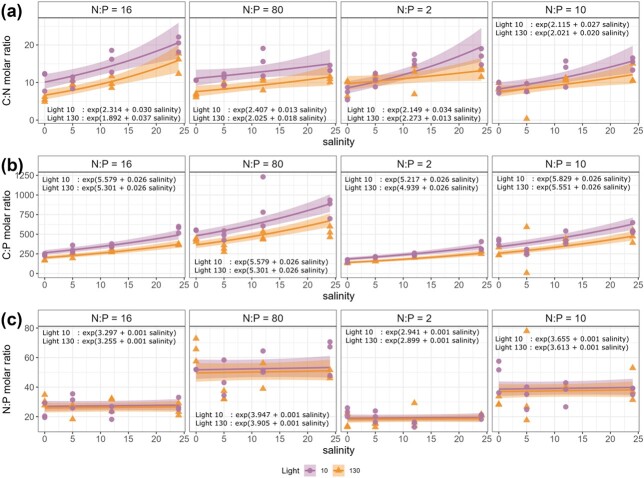
The molar ratio of (a) POC:PON, (b) POC:POP and (c) PON:POP under different combinations of salinity (0, 5, 12 and 24 psu), light (circle = 10, triangle = 130 μmol photons m^−2^ s^−1^), and nutrient supply (N:P molar ratio = 16, 80, 2 and 10, left to right panel) conditions. The lines are predicted values from the GLMs with 95% confidence intervals.

The C:nutrient ratios increased significantly with salinity (Fig. 5). Salinity explained 30% of the variation in POC:PON ratio and 17% in POC:POP ratio. The average C:nutrient ratios were higher in low-light conditions compared to high light conditions. Light intensity explained 10% of the variation in POC:PON ratio and 8% in POC:POP. The PON:POP ratio was affected only by the initial nutrient supply, which explained 71% of the variation.

## DISCUSSION

Salinity is an important environmental parameter that determines the optimal growth conditions for phytoplankton, and different species and strains can have different salinity optima and tolerance ranges (Brand, 1984; Orizar and Lewandowska, 2022). Extended exposure to lower or higher salinity outside the optimal range challenges phytoplankton cells, causing hypo- or hyperosmotic stress (Kirst, 1989). Phytoplankton responses to osmotic stress require reallocating resources to resist its harmful effect. Therefore, resource availability and acquisition are essential for phytoplankton to survive salinity fluctuations. Salinity changes can also facilitate shifts in community composition according to species-specific optima, thereby affecting the elemental composition of the phytoplankton community (Hillebrand *et al.*, 2013; Sharoni and Halevy, 2020).

First, we asked how the elemental composition of the phytoplankton assemblage varies along the salinity gradient when grown under different levels of light intensity and inorganic nutrient ratios. We determined a stronger salinity effect on POC compared to nutrient ratio and light intensity. The POC concentrations increased along the salinity gradient with a similar slope for all light and nutrient ratio conditions (Fig. 4a). A similar trend was observed from coastal phytoplankton community in the Antarctic, wherein low salinity conditions caused oxidative stress leading to a decrease in biomass (Hernando *et al.*, 2015). The decrease in POC under fresher conditions in the polyculture emphasizes the importance of salinity for phytoplankton carbon capture potential.

The PON and POP concentrations were less dependent on salinity than POC and clearly corresponded to the applied nutrient treatment, i.e. we measured low PON concentrations in N-depleted treatments and low POP concentrations in P-depleted treatments by the end of the experiment (Fig. 4). Such PON/P response is expected as phytoplankton elemental composition often reflects the type of nutrient limitation in the environment (Hillebrand *et al.*, 2013; Galbraith and Martiny, 2015). Consequently, we measured lower PON:POP ratios in N-depleted treatments and higher PON:POP ratios in the P-depleted treatment, but no significant PON:POP response to salinity change (Fig. 5c).

As a result of rising POC accumulation with salinity, POC:PON and POC:POP ratios also increased (Fig. 5). POC:PON ratios were higher under low-light conditions except at low salinities when N supply was depleted (Fig. 5). This corresponded well to the results of Fluo_max_ (Fig. S2a) and chl-*a* concentrations at the end of the experiment (Fig. S3b). Nitrogen is an essential nutrient in synthesis of pigments, and increased cellular chl-*a* content in photoautotrophs is typically needed under light limitation to maximize photon capture (Geider *et al.*, 1998; Moreno and Martiny, 2018). In our experiment, the polycultures were dominated by *P. tricornutum* (Fig. S4)*,* which has been shown to upregulate genes responsible not only for the synthesis of chl-*a,* but also accessory pigments, such as fucoxanthin, under low-light conditions (Agarwal *et al.*, 2023).

Second, we asked to what extent changes in phytoplankton stoichiometry can be explained by shifting proportions of different species in the polyculture. Intermediate salinity levels (5 and 12 psu), corresponding to the environmental conditions where all phytoplankton species were isolated, were optimal for most species (Fig. 2), and resource limitation played only a minor role in modulating phytoplankton response to salinity change. The abundance of the dominant species, *P. tricornutum,* increased with salinity (Fig. 2) and was strongly correlated with POC (Fig. S7). In contrast to *P. tricornutum*, the abundance of a green algae - *Monoraphidium* sp. declined with salinity (Fig. 2). Although green algae have a rather high N:P ratio, compared to diatoms (Finkel *et al.*, 2010; Hillebrand *et al.*, 2013), we did not observe significant change in PON:POP ratio of the polyculture along the salinity gradient corresponding to the shift in community composition (Fig. 5). Overall, community composition explained only a small proportion of the variation between experimental units, as indicated by MFA (Fig. 3). This suggests that the functioning of the polyculture and its response to salinity fluctuations were relying on the performance of the dominant *P. tricornutum*, following the classic concept of the foundation species (Dayton, 1975). As *P. tricornutum* showed euryhaline characteristics with relatively high growth rates across all salinity treatments, other species with a narrower salinity tolerance range were outcompeted, even under a limited nutrient supply. Nevertheless, small spherical species, such as a haptophyte *Diacronema lutheri,* thrived under P-depleted conditions (Fig. 2) due to their high surface area to volume ratio, which makes it easier to assimilate limited nutrients (Padisák *et al.*, 2003; Finkel *et al.*, 2010; Durante *et al.*, 2019; Ryabov *et al.*, 2021). Similarly, a small cyanobacteria *Synechococcus* sp., which, although not observed to perform nitrogen fixation, can utilize diverse forms of nitrogen (Moore *et al.*, 2002; Aldunate *et al.*, 2020), was a good competitor under N-depleted conditions (Fig. 2).

## CONCLUSIONS

Results of our experiment suggest that changes in carbon accumulation and elemental stoichiometry of phytoplankton communities along the salinity gradient were determined by the performance of the dominant species (in this study, *P. tricornutum*). In brackish areas like the Baltic Sea and estuaries, where salinity is predicted to decline in the future (Meier *et al.*, 2012; Vuorinen *et al.*, 2015; Lehmann *et al.*, 2022), low carbon accumulation by phytoplankton at low salinities emphasizes the risk of hypoosmotic stress for the functioning of coastal ecosystems and their carbon sequestration potential, as most species have higher salinity optima.

## Supplementary Material

Orizar_et_al_Revision_supplementary_fbae031

## Data Availability

The raw data supporting the conclusion of this article will be made available by the authors,without undue reservation.
