# Correction to: Phytoplankton stoichiometry along the salinity gradient under limited nutrient and light supply

**DOI:** 10.1093/plankt/fbae055

**Published:** 2024-11-03

This is a correction to: Iris D S Orizar, Sonja I Repetti, Aleksandra M Lewandowska, Phytoplankton stoichiometry along the salinity gradient under limited nutrient and light supply, *Journal of Plankton Research*, Volume 46, Issue 4, July/August 2024, Pages 387–397, https://doi.org/10.1093/plankt/fbae031

The originally published version of this manuscript has been amended to correct the labels of Figure 3C to read “LIGHT CONDITIONS”, “INITIAL NUTRIENT N:P CONDITIONS” and “SALINITY CONDITIONS”.

