# Supplementary material for: Phytoplankton stoichiometry along the salinity gradient under limited nutrient and light supply
Source: J Plankton Res. 2024 Jun 11;46(4):387–97. doi: 10.1093/plankt/fbae031 (PMC11290246; doi:10.1093/plankt/fbae031)
Supplement: Orizar_et_al_Revision_supplementary_fbae031 [file orizar_et_al_revision_supplementary_fbae031.docx]

**Phytoplankton stoichiometry along the salinity gradient under limited nutrient and light supply**

**Iris D.S. Orizar^1*^, Sonja I. Repetti^1^, and Aleksandra M. Lewandowska^1^**

**^1^**Tvärminne Zoological Station, Faculty of Biological and Environmental Sciences, University of Helsinki, Hanko, Finland

*Correspondence:

Iris D. Orizar

iris.orizar@helsinki.fi

**Supplemental Figures**


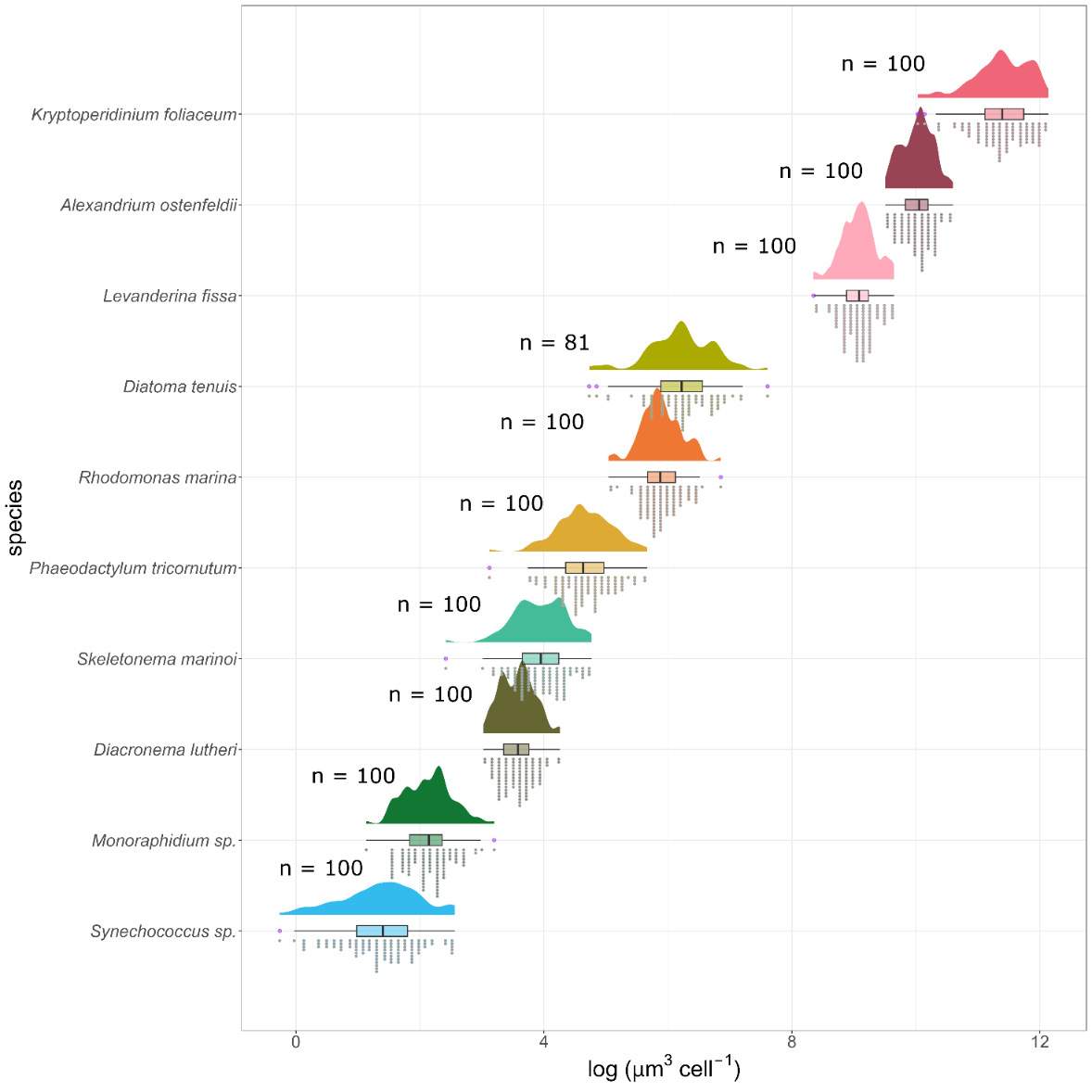


**Fig. S1.** Calculated biovolume (density and box plot) of the species from the stock cultures. The shape and formula used for calculating the species-specific biovolume was based on Olenina et al. 2006.


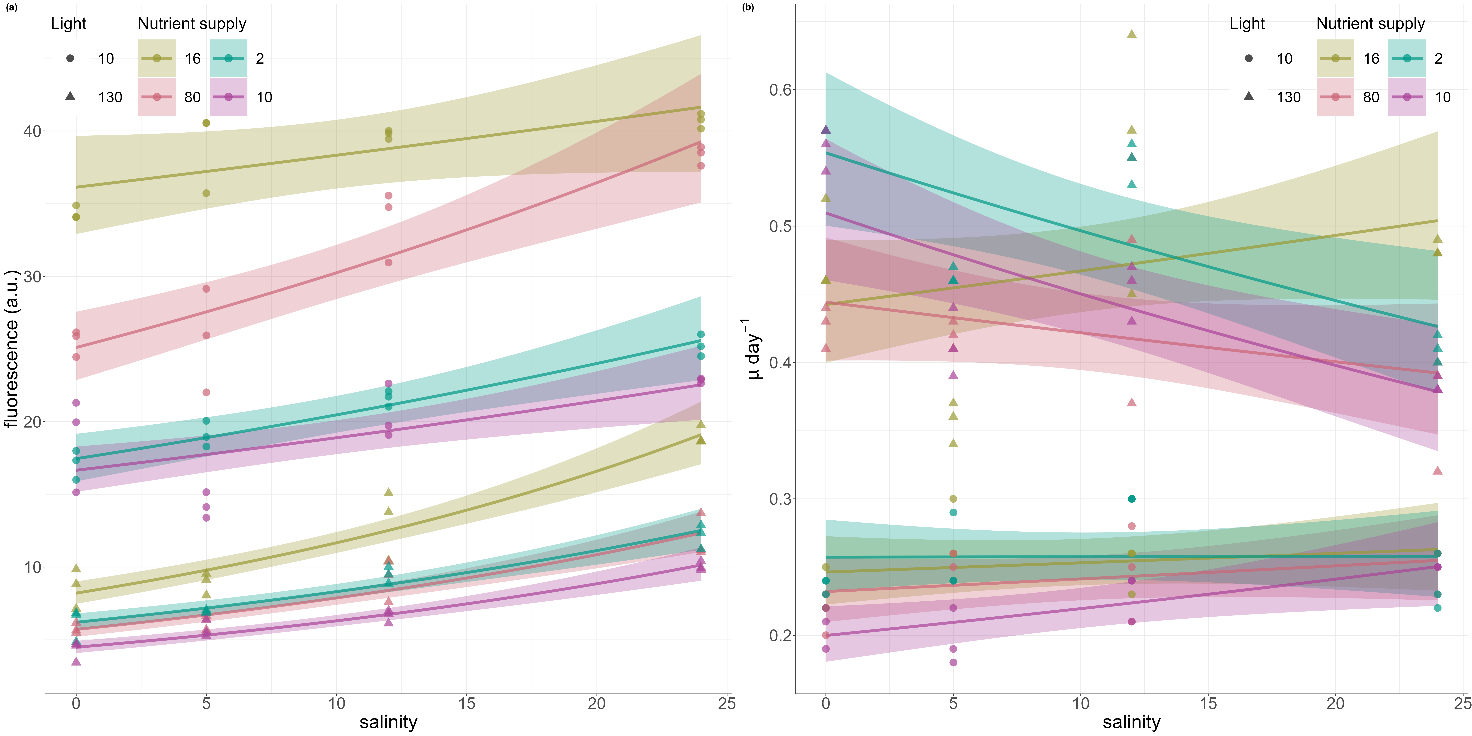


**Fig. S2.** (a) Maximum chl-*a* fluorescence (Fluo_max_) and (b) growth rate (µ) of the polyculture along the salinity gradient under different light (shape: µmol photons m^-2^ s^-1^) and nutrient ratios (color: nitrogen to phosphorus molar ratio). The smoother lines are predicted values based on the glm model: *response variable ~ salinity * light * nutrient supply*. Ribbon = 95 % confidence interval.


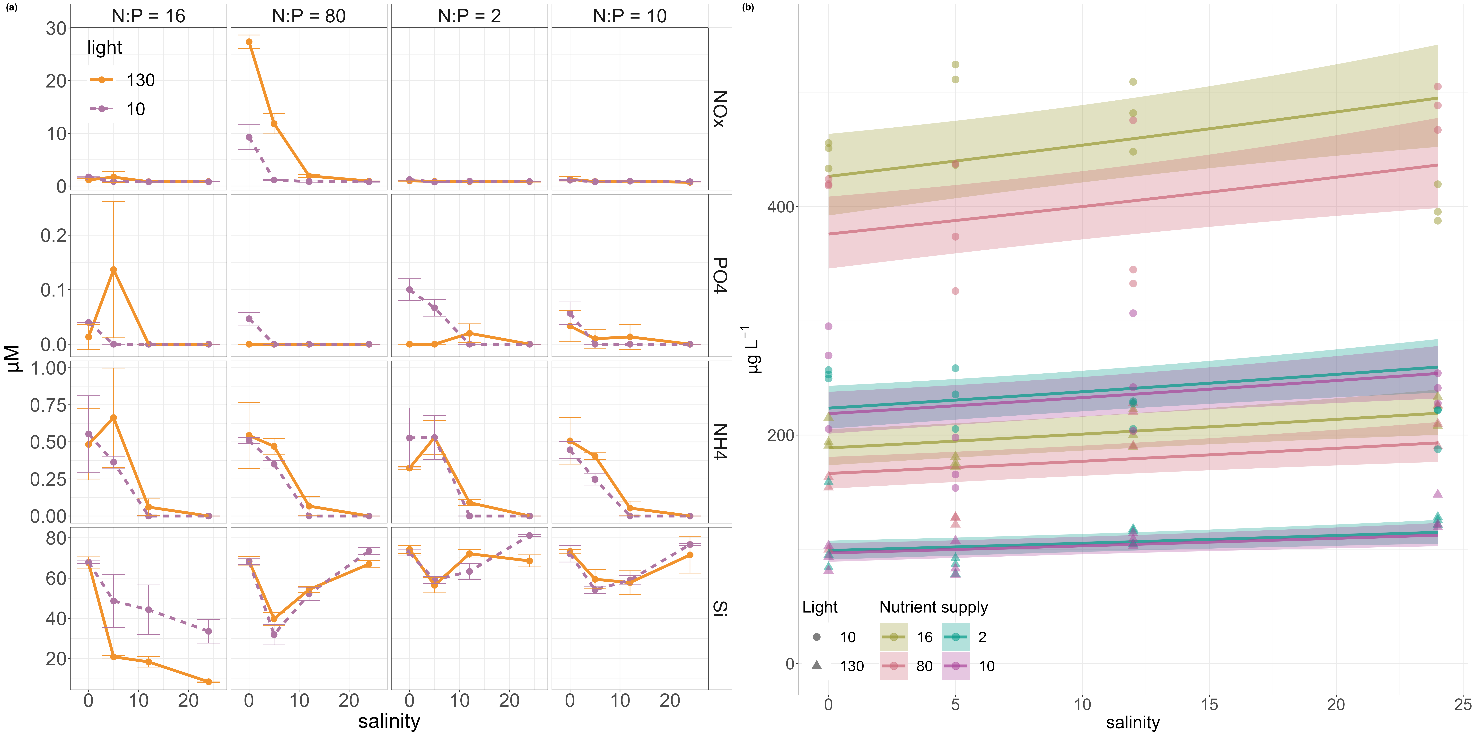


**Fig. S3. (a)** Concentration of the residual nutrients at the end of the incubation periods for each treatment. Error bar = standard deviation (n = 3). Columns = nutrient supply conditions N:P molar ratio; rows = different inorganic nutrients. **(b)** Total chlorophyll *a* concentration along the salinity gradient. Shape = light intensity (µmole photons m^-2^ s ^-1^); Color = nutrient ratios N:P molar ratio. Solid line and ribbon = predicted values based on the glm model: *total chl ~ salinity + light + salinity*. Ribbon = 95 % confidence interval.


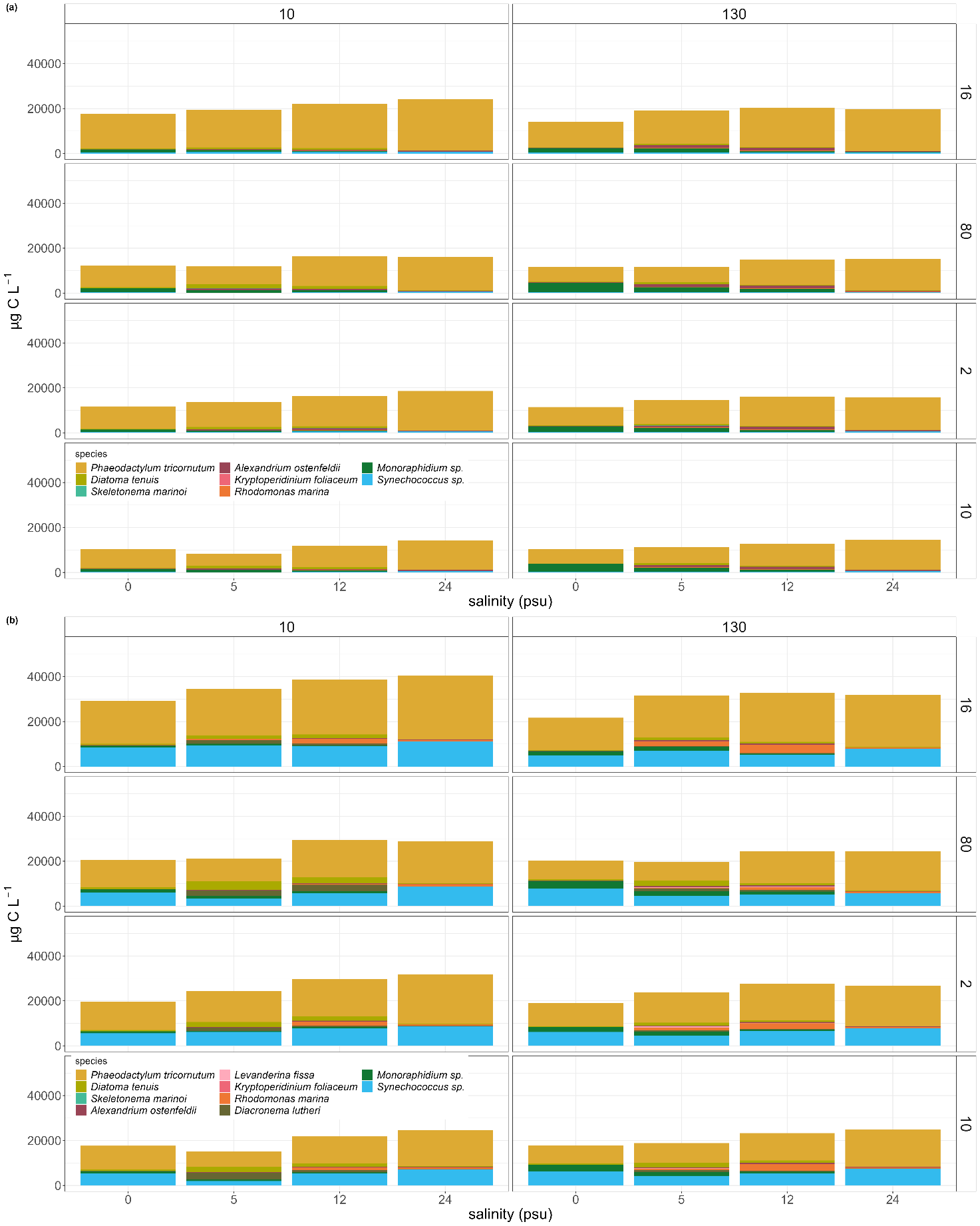


**Fig. S4.** Calculated particulate organic carbon content for each of the species in the polyculture at the end of the incubation period. Rows = nutrient supply conditions N:P molar ratio; Columns = light intensity (µmole photons m^-2^ s ^-1^). (a) Estimated POC based on Olenina et al. 2006. Data for *L. fissa* and *D. lutheri* were not available, hence not excluded from the figure. (b) Estimated POC based on Orizar and Lewandowska unpublished data for individual species. The black dots are the actual POC concentration measured from the polyculture in each treatment (n = 3).


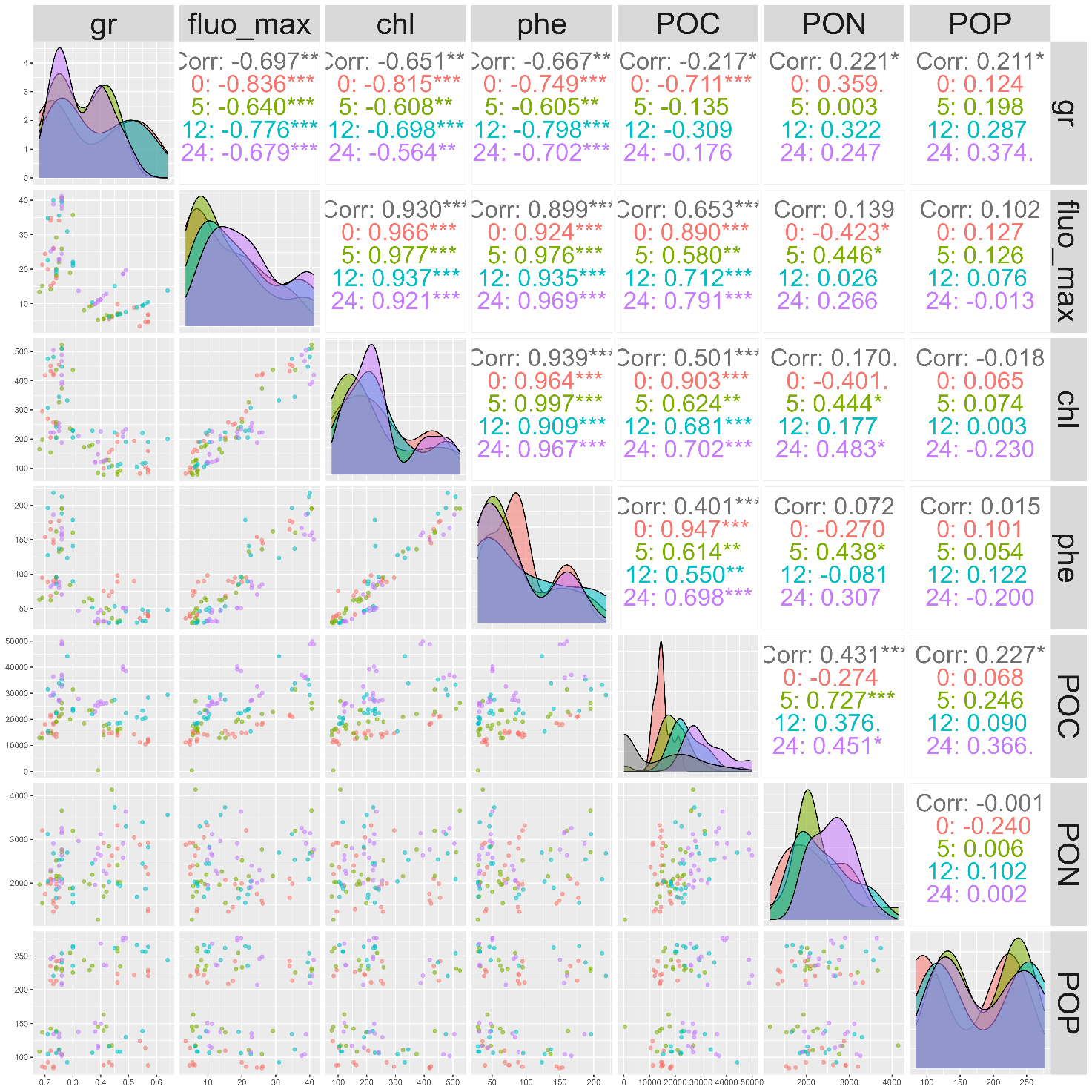


**Fig. S5.** Pearson correlation coefficient of the different traits measured from the polycultures grouped by salinity conditions. gr = growth rate (µ), fluo_max = maximum chl *a* fluorescence, chl = chl *a* concentration, phe = pheophytin *a* concentration, POC = particulat organic carbon, PON = particulate organic nitrogen, POP = particulate organic phosphorus.


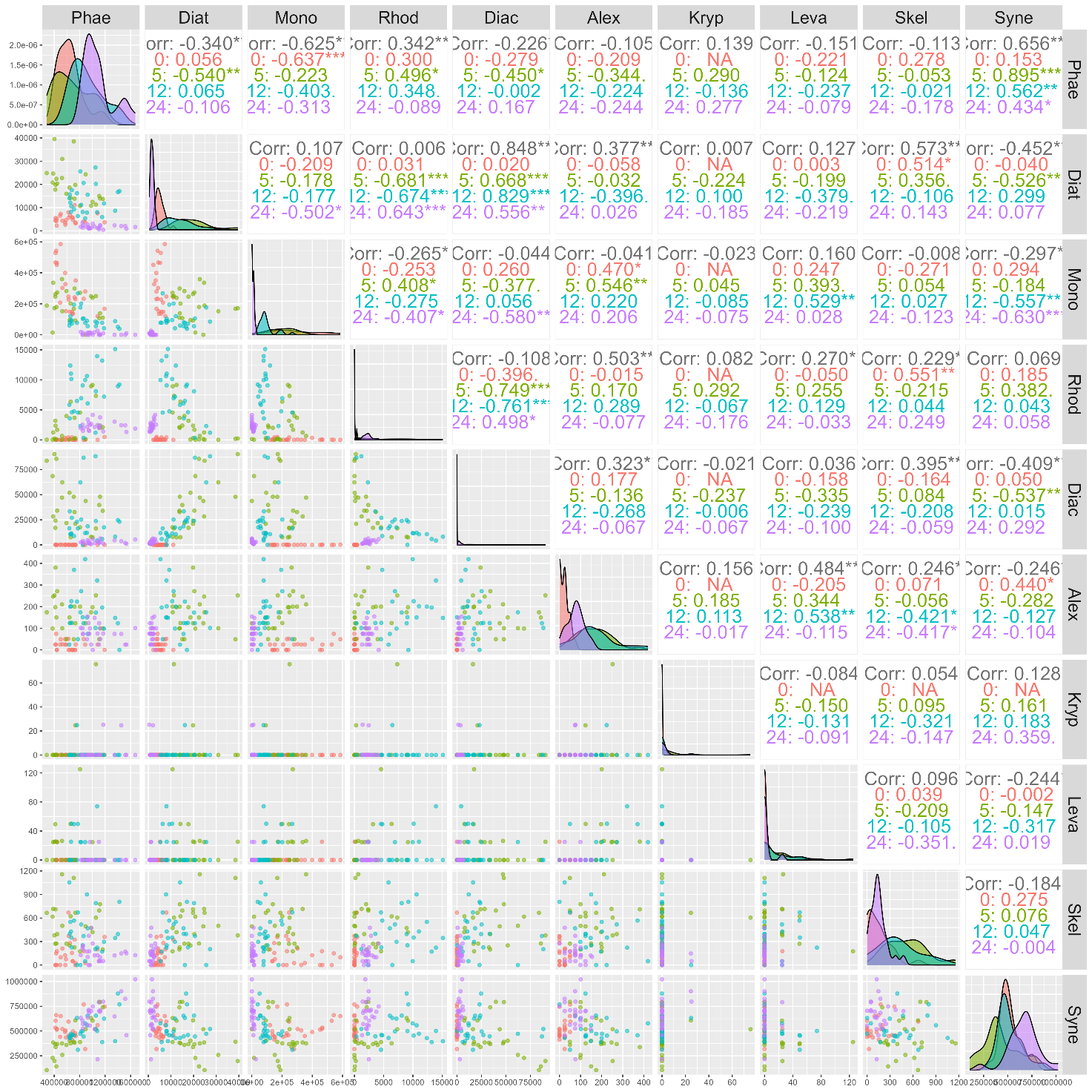


**Fig. S6.** Pearson correlation coefficient with scatter plot and histogram between pairs of the species in the polyculture. Phae = *Phaeodactylum tricornutum*, Diat = *Diatoma tenuis*, Skel = *Skeletonema marinoi*, Alex = *Alexandrium ostenfeldii*, Kryp = *Kryptoperidinium foliaceum*, Leva = *Levanderina fissa*, Rhod = *Rhodomonas marina*, Mono = *Monoraphidium* sp., Diac = *Diacronema lutheri*, Syne = *Synechococcus* sp.


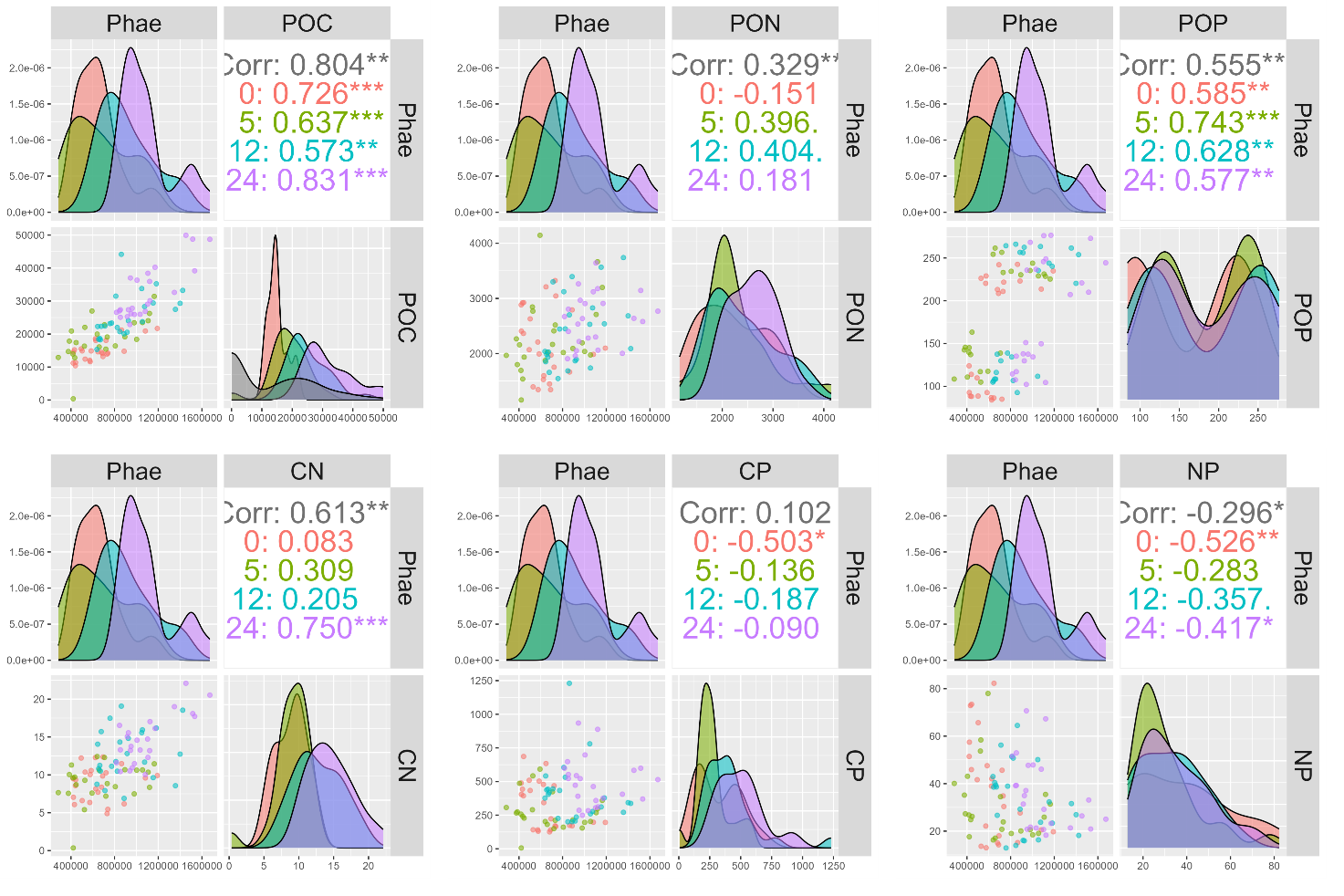


**Fig. S7.** Pearson correlation coefficient with scatterplot and density plot between *Phaeodactylum tricornutum* (Phae) abundance and particulate organic C, N, and P concentrations and ratios.


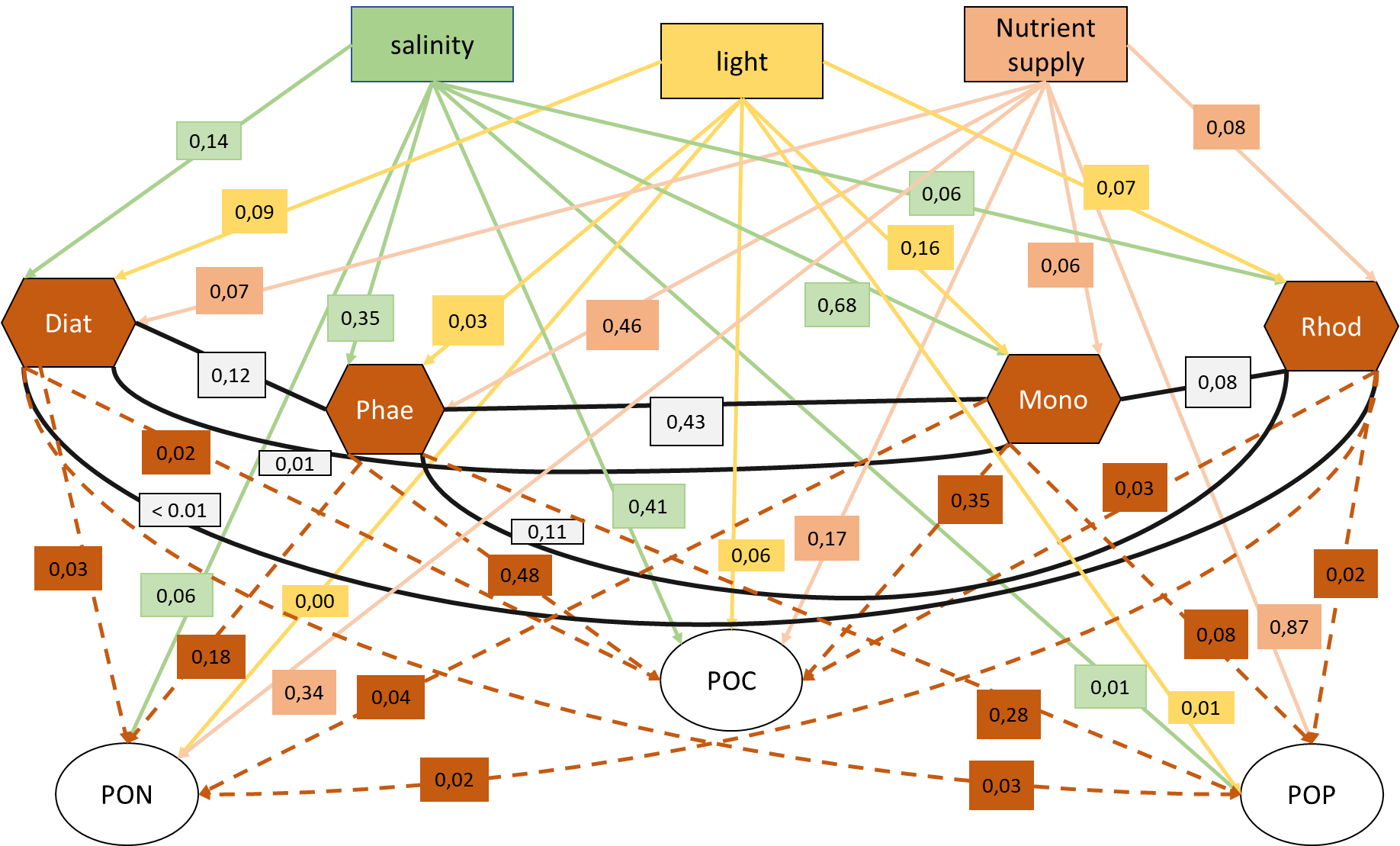


**Fig. S8.** Schematic showing the effect of environmental parameters (salinity, light, and nutrient supply) on *Diatoma tenuis* (Diat), *Phaeodactylum tricornutum* (Phae), *Monoraphidium* sp. (Mono), and *Rhodomonas marina* (Rhod), and particulate organic carbon, nitrogen, and phosphorus. The numeric values are the McFadden’s R square based on the general model *glm (response ~ effect)*
